# The Influence of Substrate on the Optical Properties of Gold Nanoslits

**DOI:** 10.3390/jimaging9120269

**Published:** 2023-12-03

**Authors:** Ammara Ammara, Ghulam Abbas, Francesco V. Pepe, Muhammad Afzaal, Muhammad Qamar, Abdul Ghuffar

**Affiliations:** 1 Dipartimento Interateneo di Fisica, Università degli Studi di Bari Aldo Moro, I-70126 Bari, Italy; ammararana53@gmail.com (A.A.);; 2 Department of Physics, Riphah International University, Faisalabad Campus, Faisalabad 38000, Pakistan; 3 Department of Physics, Ghazi University, Dera Ghazi Khan 33401, Pakistan; 4 Istituto Nazionale di Fisica Nucleare, Sezione di Bari, I-70125 Bari, Italy

**Keywords:** field enhancement, optical properties, finite element method

## Abstract

Nanoslits have various applications, including localized surface plasmon resonance (LSPR)-based nanodevices, optical biosensors, superfocusing, high-efficiency refractive index sensors and chip-based protein detection. In this study, the effect of substrates on the optical properties of gold nanoslits placed in free space is discussed; for this purpose, glass BK7 and Al${}_{2}$O${}_{3}$ are used as substrates and the wavelength of incident light is supposed to be 650 nm. The optical properties, power flow and electric field enhancement for gold nanoslits are investigated by using the finite element method (FEM) in COMSOL Multiphysics software. The effect of polarization of an incident electromagnetic wave as it propagates from a gold nanoslit is also analyzed. As special case, the effect of glass and alumina substrate on magnetic field, power flow and electric field enhancement is discussed. The goal of this research is to investigate the phenomenon of power flow and electric field enhancement. The study of power flow in gold nanoslits provides valuable insights into the behavior of light at the nanoscale and offers opportunities for developing novel applications in the field of nanophotonics and plasmonics. The consequences of this study show the significance of gold nanoslits as optical nanosensors.

## 1. Introduction

Nanoparticles of noble metals like gold, silver and copper show optical properties that are generally different from their bulk materials, due to their nano size and surface effects. In nanomaterials, individual particle morphology fixes their physical, chemical and even biological properties [1,2]. Metals’ optical properties are indicated in terms of two physical quantities: the refractive index and the absorption coefficient. Gold has a special significance in the field of metal optics, due to its practical use in optoelectronic and nano-optical systems. It is also used as a model system for the investigation of the fundamental electronic excitations that increase electromagnetic field interactions in metals [3,4,5]. When gold nanoparticles are stimulated by incident light, they interact significantly with light and the conduction electrons on the metal surface oscillate collectively. Because of surface plasmon resonance occurrences, they have adjustable and distinctive optical characteristics [4,6]. A series of well-established techniques can be used to synthesize gold and silver nanoparticles [7,8,9].

The phenomenon of extraordinary optical transmission (EOT), observed in periodic arrays of subwavelength holes [4,10,11] or slits [12,13,14,15,16,17], has garnered significant attention. Novel spoof surface plasmon polaritons (SPPs) in perfect electrical conductors have been identified as an alternative mechanism for achieving EOT, extending its applicability beyond transverse magnetic (TM) modes [18,19,20]. Initially attributed to the presence of surface plasmon polaritons (SPPs), which are predominantly excited by transverse magnetic (TM) waves, recent studies have demonstrated the possibility of EOT for transverse electric (TE) waves as well [21,22,23,24,25,26]. Notably, some researchers have successfully realized EOT in configurations that accommodate both TM and TE waves at the same wavelength, as supported by theoretical analyses, numerical simulations, and experimental observations [27,28,29]. Gold nanoparticles (Au-NPs) exhibit low optical frequency loss and strong electrical conductivity during the propagation of surface plasmons behaving like plasmonic materials when their real part of electric permittivity is negative. The chemical, physical, and optical characteristics of Au-NPs are well known [30,31].

Surface plasmons (SPs) [32] are particularly suited for the detection of surface binding events because these are highly responsive to the dielectric constant when close to the surface. Localized surface plasmon resonance (LSPR), which is induced by resonant SPs localized in nanostructures, can be applied to scanning near-field microscopy with chemical resolution [33] and the detection of single chemical or biological agent molecules [7]. These nanoslit structures can be used in different applications, such as plasmonic lenses, by tuning the slit width and refractive index [34]. Other potential applications of nanoslits are represented by waveguides [35], filters [36], gratings [37] and sensors [38], which can be used in devices able to control the propagation of light.

Metallic structures at the nanoscale have generated significant interest, since many studies have confirmed that light transmitted by a subwavelength hole array is more than expected from the classical theory of diffraction [6,8,39] and linked EOT to the SP resonant excitation caused by the the array periodicity [40]. The application of nanostructured arrays in biological detection and surface-based chemicals is driven by their distinctive optical properties in transmission surface plasmon resonance (T-SPR).

The material on which processing is accomplished is referred to as the substrate in the fields of engineering and materials science. To generate new films or material layers, such as deposited coatings, one might use this surface. A typical substrate for coating deposition could be hard like metals, ceramic, or crystal. However, flexible substrates are used as well.

The content of the article is the following. In Section 2, the formulation for plane wave propagation inside the slit is discussed. In Section 3, the optical properties of gold nanoslits and the effect of substrates are discussed in detail.

## 2. Formulation

**Figure 1:**
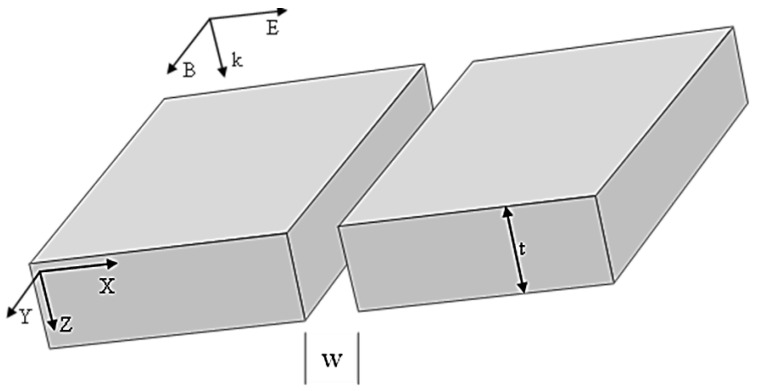
Representation of a nanoslit characterized by thickness *t* and width *w*. The nanoslit is located in free space. The vector k represents a plane wave, polarized in a transverse magnetic (TM) mode, with normal incidence on the top surface.

The geometry of a gold nanoslit model is shown in Figure 1; it is assumed to be infinitely extended along the *y* axis, and characterized by thickness *t* and width *w*. The gold nanoslit is placed in lossless, nondispersive and homogeneous surrounding medium, with the same properties as free space.

### 2.1. Permittivity and Conductivity

Our results are based on the Maxwell’s equations

(1) $$\begin{array}{cc} \nabla \cdot E & =0, \end{array}$$

(2) $$\begin{array}{cc} \nabla \cdot B & =0, \end{array}$$

(3) $$\begin{array}{cc} \nabla \times E & =-\frac{\partial B}{\partial t}, \end{array}$$

(4) $$\begin{array}{cc} \nabla \times B & =\mu \varepsilon \frac{\partial E}{\partial t}+\mu J_{c}, \end{array}$$

with E and B the electric and magnetic field, respectively, and the conduction current is determined by the electric field as $J_{c}=\sigma E$, with σ being the material’s conductivity, while ε is the dielectric permittivity and μ the magnetic permeability. Applying the curl technique to above equations first decouples the Maxwell’s equations as

(5) $$\begin{array}{cc} \nabla^{2}E & =\mu \varepsilon \frac{\partial^{2}E}{\partial t^{2}}+\mu \sigma \frac{\partial E}{\partial t}, \end{array}$$

(6) $$\begin{array}{cc} \nabla^{2}B & =\mu \varepsilon \frac{\partial^{2}B}{\partial t^{2}}+\mu \sigma \frac{\partial B}{\partial t}. \end{array}$$

The obtained expressions represent wave equations describing the electromagnetic wave propagation in the metal. Let us focus on plane-wave solutions [41], characterized by the form

(7) $$\begin{array}{cc} E_{m}\left(r,t\right) & ={\tilde{E}}_{0,m}\exp\left(i({\tilde{k}}_{m}z-\omega t)\right), \end{array}$$

(8) $$\begin{array}{cc} B_{m}\left(r,t\right) & ={\tilde{B}}_{0,m}\exp\left(i({\tilde{k}}_{m}z-\omega t)\right), \end{array}$$

where the complex wavenumber ${\tilde{k}}_{m}$ can be expressed as ${\tilde{k}}_{m}=k_{0}\tilde{n}=k_{0}\left(n_{1}+in_{2}\right)$, in terms of the wavenumber $k_{0}$ in free space and a complex refraction index $\tilde{n}=n_{1}+in_{2}$ [42], while ${\tilde{E}}_{0,m}$ and ${\tilde{B}}_{0,m}$ represent the complex amplitudes of the field in metal. The fields in Equations (7) and (8) provide a plane-wave basis, which can be used to construct arbitrary superpositions that propagate in the metal at different frequencies and in different directions. The amplitude distribution of these plane wave components is called the “angular spectrum” of the field [43]. Plugging the plane-wave solutions into the propagation equations provides expressions for the propagation parameter in terms of metal permeability, complex conductivity, and complex permittivity. The real and imaginary components of the complex permittivity can be expressed as

(9) $$\varepsilon_{1}=\varepsilon^{\prime }-\frac{\sigma_{i}}{\varepsilon_{0}\omega },\;\varepsilon_{2}=\varepsilon^{\prime \prime }+\frac{\sigma_{r}}{\varepsilon_{0}\omega },$$

where the terms $\varepsilon^{\prime }$ and $\varepsilon^{\prime \prime }$ represent the metal dispersive and absorptive responses without the conduction electrons’ contribution, while the terms depending on $\sigma_{i}$ and $\sigma_{r}$ provide the contribution of the conducting electrons’ permittivity.

The relations among field phases and amplitudes at the metal/dielectric boundary are the following:

- The phase of the electric field in the metal is retarded by π/2 with respect to the magnetic field phase in the metal.
- The amplitude of the electric field incident at the surface is reduced by a factor of $2k_{0}/\left|{\tilde{k}}_{m}\right|$ and lags the electric field phase in the metal by π/2.
- The magnetic field in the metal has an amplitude that is roughly twice than that of the incident field.
- The frequency is constant across the boundaries.
- The conduction current $J_{c}$ is in phase with the incident electric field, and the reflected electric field is out of phase by π with respect to the incident electric field.

### 2.2. Electromagnetic Fields Generated Inside the Nanoslit

The presence of currents $J_{p}$ and $J_{spp}$ in the metal skin is due to Faraday induction, resulting from the fact that the magnetic field tends to form surface standing waves. These conduction currents lead to the charging of the edges of the slit, resembling a standard capacitor with parallel plates. Moreover, the displacement current $J_{slit}$, flowing through the slit gap, produces oscillating E and B field components that are in quadrature with the incident plane wave. Initially, let us examine the characteristics of the slit fields induced by the plane standing wave. Since ∇·J = 0, based on the continuity of currents at the slit gap [42],

(10) $$J_{p}=J_{slit}=\varepsilon_{0}\varepsilon_{slit}\frac{\partial }{\partial t}E_{slit}$$

The plane wave-induced fields in the slit are

(11) $$\begin{array}{cc} E_{slit}^{p} & =i{\left(\frac{\omega_{p}}{\omega }\right)}^{2}\frac{2E_{I}}{n_{2}\left|\varepsilon_{slit}\right|}, \end{array}$$

(12) $$\begin{array}{cc} B_{slit}^{p} & =i{\left(\frac{\omega_{p}}{\omega }\right)}^{2}\frac{2E_{I}}{n_{2}\left|\varepsilon_{slit}\right|c}, \end{array}$$

with $\omega_{p}$ being the bulk plasmon frequencies and $E_{I}$ being the incident electric slit amplitude. We can observe that the magnetic field in the slit is in quadrature with the incident electromagnetic field. Thus, such fields are associated with the slit gap propagation mode. We consider the fields produced on the surface and in the slits by the identical standing wave.

## 3. Results

The COMSOL Multiphysics software was used to numerically model the gold nanoslit. The numerical modeling procedures in the FEM were carried out in this order: The issue was divided into a set of smaller portions, the prevailing equations for each component first established, and then the system of all equations was solved. The RF module in COMSOL Multiphysics was used to run the numerical simulations and to study the proposed 2D geometry of the problem. We chose the value of V/m for the background electric field. For gold nanoslits, we fixed the slit width to 100 nm and the height to 500 nm, with incident light of wavelength 650 nm. Unwanted reflections from the system boundaries were avoided through an appropriate choice of boundary conditions. We characterized the obtained results through the electric and magnetic field as well as power flow for the transverse magnetic or p-polarization. Data from Johnson and Christy [31] were used to model the dielectric properties of gold. Moreover, the optical properties of a gold nanoslit were studied using the appropriate boundary conditions.

### 3.1. Electric Field Enhancement

The most fundamental plasmonic structure for understanding electromagnetic field confinement between metallic nanostructures is a thin metallic slit, according to elementary physics principles. Parameters used in this work are listed in Table 1.

#### 3.1.1. The Cutoff for Subwavelength Slits under $E_{\|}$ (TE) Illumination

We first evaluated field distributions for a subwavelength slit (W=100) under $E_{\|}$ illumination. For $E_{\|}$, the propagation of fields through the nanoslit is zero because the boundary conditions are not satisfied [30]. At the top air-to-gold interface, the magnetic field $H_{y}$ is maximum, while the total electric field $E_{x}^{total}=E_{x}^{inc}+E_{x}^{ref}$ in this region is zero. This is the reason why a very small amount of electric field is required to start moving the conduction electrons in order to produce the surface current $J_{x}$ at the top surface of the conductor. Just above the conductors, the magnetic field component $H_{y}$ is determined by the surface current according to Ampère’s law. $E_{x}$ must be continuous at the metal interface, and negligible in the bulk, considering Maxwell’s equations’ requirements. Eventually, we find an interface value $E_{x}^{total}≃0$.

Because the boundary conditions do not match, when the incident wave comse from the top and travels parallel, there will be no light passing through the slit, and no propagation of light was observed in the simulation. In the case of $E_{\|}$, transmission of light through the nanoslit does not occur, regardless of the slit width [39]. This case does not depend upon the parameters. As a result, the output is zero and there is no amplitude enhancement of the electric field for the transverse electric mode (TE). The magnetic field present at the surface is maximum, while the total electric field is zero in this region.

#### 3.1.2. $E_{⊥}$ (TM) Light Transmission through Subwavelength Slits

**Figure 2:**
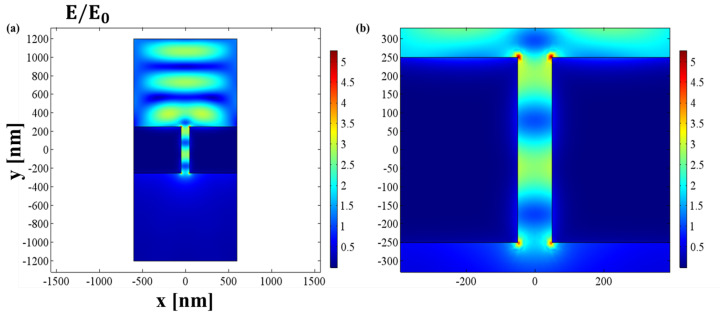
Gold nanoslit of width w=100nm in a metallic structure of thickness t=500nm: (**a**) electric field distribution, with the color bar in V/m; (**b**) extended view of electric field inside the nanoslit under p-wave illumination at the wavelength λ=650nm, with the color bar in V/m.

We now explore the phenomenon of field enhancement. Figure 2 shows computed plots of electric and magnetic fields for a 100 nm wide slit in a 500 nm thick gold film. Transmission is supposed to be caused by strong electric fields and magnetic fields propagating along the slit walls, which are supported by the currents and proper distribution of surface charges on gold nanoslit walls. On the top surface, a very small $E_{y}$ is required to sustain the surface current $J_{y}$, resulting in the magnetic field component $H_{x}$ being close to the surface. Due to $J_{y}$ stopping abruptly at the slit edge, where the (oscillating) charges on the opposing sides of the slit behave as an electric dipole, charges build up at the corners of the slit. The electric dipole created by the (oscillating) charges on the opposite slit edges plays a part in enhancing transmission through the slit at the surface of the metal. The continuity equation ∇·J+∂ρ/∂t=0 relates the charge density with the current density J.

The incident plane wave creates a second electric dipole when it reaches the bottom of the slit, which results in an upward-moving wave inside the slit. The charges and currents are rearranged by the counter-propagating wave into a standing wave in accordance with the interference pattern between the electromagnetic fields. Charges build up on each side of the slit in the lower corners, the wall’s center, and the upper corner. However, the wall and upper surface of the slit are also subject to strong currents, and charges are also present on the right and left sides of the slit, and they display opposing signs. A stationary wave (standing wave) forms inside a material with a magnetic field that is close to the conductor as a result of an electromagnetic plane wave that is incident on and reflected from the dielectric-metal interface. If a slit is present into the metal surface, one observes the establishment of a displacement current in the dielectric gap, and of oscillating charge dipoles at the edges, in quadrature phase with incident field. As they radiate into the aperture, these dipoles are releasing surface waves from their edges.

There is an electric dipole at the sharp edges of the slit because the current J stops there, allowing charges to build up at the sharp corners and acting as an electric dipole when they oscillate on the opposite ends of the slit. When the incident wave in TM mode in Figure 2a comes from the top and travels perpendicularly (downward) before hitting the interface, a standing wave is created above the interface as a result of the interference between the incident and reflected electric and magnetic fields. In Figure 2a, these electric dipoles may be seen at the top and bottom. It is believed that these dipoles, when combined with the surface plasmon at the metal–dielectric–metal contact, contribute to the field enhancement. The traveling beam is carried downward by surface charges and currents inside the waveguide. A second electric dipole, depicted in Figure 2b, is created when this traveling beam ranges to the bottom of the waveguide and finds an impedance mismatch. This powerful dipole causes an upward traveling wave to be produced inside the waveguide. Inside the waveguide, a standing wave is produced by the opposing traveling waves. In Figure 2, we see a maximum enhancement in the value of field up to 5 times. These results demonstrate that the restricted and dipolar characteristics of the resonance are clearly revealed by the pattern of the electric field amplitude at the top surface starts of the slit. The electric field enhancement obtained in this case can be relevant to increase sensitivity, which is one of the key factors for surface-plasmon-resonance-based optical biosensors.

### 3.2. Magnetic Field

**Figure 3:**
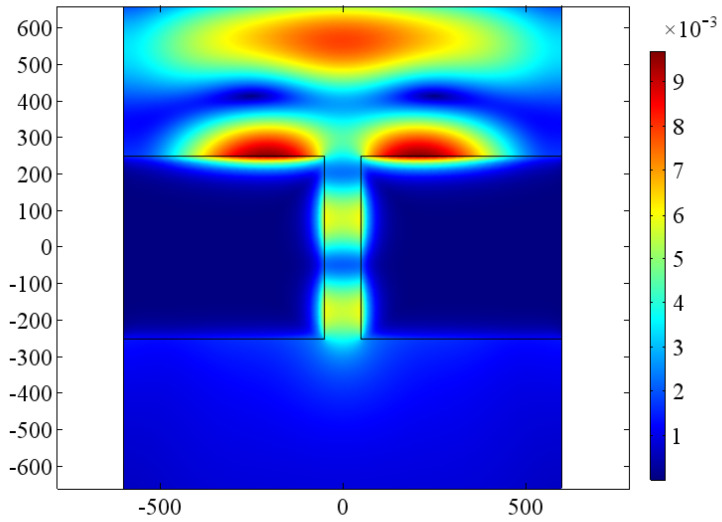
Magnetic field of a gold nanoslit of width w=100nm in a metal structure of thickness t=500nm at λ=650nm. Color bar in A/m.

**Figure 4:**
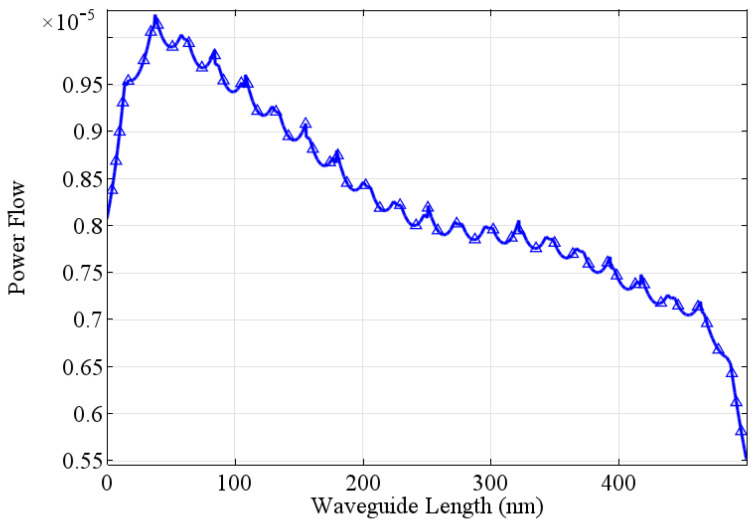
Power flow under p-wave illumination at λ=650nm gold nanoslit of width w=100nm in a metallic structure of thickness t=500nm.

A planar wave in a TM mode, incoming normal to the top surface of the gold nanoslit, is totally reflected by an ideal metal, but in the case of finite conductivity, part of the incident wave reaches to the depth. Closer to the metal surface, the magnetic field predominates, aside from the fact that the magnetic field dominates the energy density on the dielectric side of the contact and the field amplitudes (on the metal side, due to dispersion of the material). Energy density, on the other hand, is almost same in inputs from electric and magnetic field parts. The electric and magnetic fields in the slit gap and the electric and magnetic fields within the metal are determined by integrating the Maxwell–Faraday and Ampère–Maxwell equations. The fields in the gap oscillate in phase with the corner charge dipoles and are in quadrature with the incident electromagnetic field. The necessary condition for the propagation of light is that there should be a dielectric–metal interface and both media must have different refractive index. According to continuity condition, the magnetic field across the interface should be continuous. So, magnetic field is continuous at the boundary; it can be observed in Figure 3. To start wave propagation phenomena, a small amount of magnetic field is necessary at the interface. A very small amount of electric field exists at the metal–dielectric interface, and this electric field is sufficient to support the surface current, which in turn sustains the magnetic field above the surface, as demonstrated in Figure 4, which also justifies the continuity conditions of magnetic field across the interface. The maximum value of the magnetic field is $9\times 10^{-3}\;V/m$, as shown in Figure 3.

### 3.3. Power Flow

The power flow through gold nanoslits can be computed by evaluating the Poynting vector. Such nanoslits exhibit unique optical properties, including enhanced light transmission and confinement of light at nanoscale dimensions. When a plane wave of light interacts with a gold nanoslit, a portion of the incident power is transmitted through the slit while the rest is either reflected or absorbed. The power flow in gold nanoslits is highly dependent on the geometrical parameters of the structure, such as the width, height of the slit and the surrounding medium. Due to the interaction between the incident light and the nanoslit structure, a phenomenon known as surface plasmon polariton (SPP) resonance occurs. SPPs are collective oscillations of electrons at the metal-dielectric interface and can significantly impact the power flow in the nanoslit. At specific wavelengths, the SPPs are excited and can propagate along the metal surface, resulting in enhanced transmission of light through the nanoslit. This enhanced transmission is attributed to the localization and concentration of electromagnetic energy within the nanoscale aperture [1].

In this section, the phenomenon of power flow is explored. For this purpose, the same geometrical parameters are used given in Table 1. When the plane wave propagating from top (air) reaches the air–metal interface, as considered in the situation in Figure 2, most of power is reflected to air and only small part of incident power is transmitted to the metal as the skin depth is only a few nanometers so the energy flux or the power flow penetrates only a few tens of nanometers.

Figure 4 represent the power flow inside the waveguide. Fabry–Perot resonances developed inside the waveguide and metal losses reduce power flow inside the waveguide. The Fabry–Perot resonances are affected by the width of the slit. Resonance peaks in power flow appear in Figure 4, where it is also observed that the power flow inside the slit decreases when the length of the waveguide increases. The power flow in gold nanoslits is influenced by the loss mechanisms within the gold material itself. Gold is not a perfect conductor, and it exhibits intrinsic material losses that lead to absorption and dissipation of the incident power. These losses can affect the overall power transmission through the nanoslit and lead to a decrease in the transmitted power. In this case, the maximum power that flows from the nanoslit is $1.5\times 10^{-5}\;W/m^{2}$.

### 3.4. Effect of Substrate on the Transmission of Light

This section aims to investigate and analyze the effect of substrate properties on the transmission of light. The interaction between light and different substrate materials has significant implications in various fields, including optics, photonics and material science. By examining the transmission characteristics of light through distinct substrates, we can gain valuable insights into their optical properties and their potential applications. In this research, we systematically explore the impact of substrate composition, thickness, and refractive index on light transmission, employing theoretical methodology. The results provide a comprehensive understanding of the relationship between substrates and light transmission, facilitating the design and development of advanced optical devices and materials. A substrate refers to a material or surface upon which something is deposited or built. In the context of optics and light transmission, a substrate specifically refers to the material on which light propagates or passes through. It serves as the medium through which light travels. Substrates are essential components of SPR-based optical biosensors.

**Figure 5:**
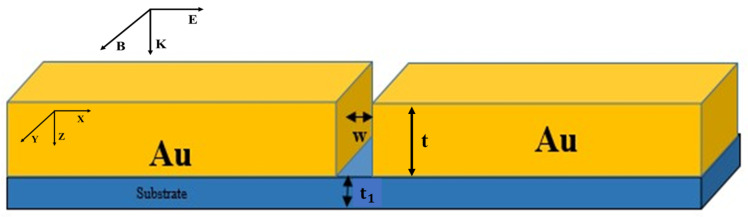
Schematic representation of a gold nanoslit placed on a substrate of thickness $t_{1}$, with an incident TM plane wave, normal to the top surface.

The use of substrate is important also because the waveguide-type structure can be fabricated on it, by adding bias between the microstrip line and the ground plane, as shown in Figure 5. Surface engineering procedures rely heavily on the substrate. It acts as a mechanical support for the surface layer, and can also interact with the latter during processing.

#### 3.4.1. Effect on Electric Field Enhancement and Power Flow of Using BK7 Glass as a Substrate

The use of BK7 glass as a substrate can have a significant effect on electric field enhancement and power flow in various applications. BK7 is a commonly used optical glass with desirable optical properties, making it suitable for numerous optical and photonic devices. The dielectric properties of BK7 were modeled using data from Schott. BK7 is known for high transmission and its clear and colorless appearance. Core applications of BK7 include lenses, windows and prisms. It is used in different applications because it is a cost-effective choice. Its low dispersion and high refractive index make it suitable for different optical systems operating in the visible region [44,45,46].

When a beam of light passes through a glass substrate like BK7, it experiences a change in its speed and direction due to refraction. This change in the propagation medium alters the electric field distribution of the light wave. In certain cases, this refraction can result in an enhanced electric field at the interface between the glass substrate and the surrounding medium. The electric field enhancement occurs because of the impedance mismatch between the two media, leading to a concentration of electric field energy in the vicinity of the interface. This effect can be advantageous in applications such as surface-enhanced spectroscopy, nonlinear optics, and plasmonics. Using BK7 as a substrate can influence the power flow in optical systems. When light propagates through a medium with a different refractive index, such as BK7, there is a change in the velocity and direction of the light wave. This change affects the power flow of the electromagnetic field. Different field enhancement and power flow for different thicknesses of substrate are given in Table 2.

#### 3.4.2. Effect on Electric Field Enhancement and Power Flow of Using Alumina (Al${}_{2}$O${}_{3}$) as a Substrate

The use of alumina (Al${}_{2}$O${}_{3}$) as a substrate material can have a positive impact on electric field enhancement and power flow. Its high dielectric constant, breakdown strength and thermal conductivity contribute to improved energy transfer, reduced power losses and reliable operation in various electronic and electrical devices. To model the dielectric properties of alumina, data from Ref. [45] were used. Alumina is naturally transparent in the ultraviolet-to-infrared region and can be used in versatile applications such as lens construction. Due to its high refractive index, alumina is known for its strong dielectric properties. It can be also used as substrate material for thin-film optical coatings.

When alumina is used as a substrate material, it can enhance the electric field in several ways. First, alumina has a high dielectric constant, which means it can store a large amount of electrical energy when an electric field is applied. This property allows for increased field strength and more efficient energy transfer in the device. Second, the high breakdown strength of alumina ensures that it can withstand high electric fields without experiencing electrical breakdown or failure. This characteristic is crucial in high-power applications where electric fields can be intense. By preventing breakdown, alumina helps maintain a stable electric field and ensures the integrity and longevity of the device. Alumina’s excellent electrical insulation properties prevent leakage currents and minimize power losses due to parasitic capacitance or undesired pathways for current flow. As a result, more power can be effectively channeled through the intended circuitry, enhancing overall device efficiency.

**Figure 6:**
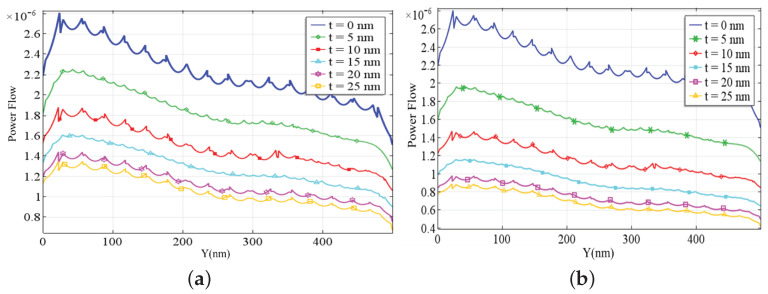
Power flow (in $W/m^{2}$) under p-wave illumination at the wavelength λ=650nm for a gold nanoslit with thickness t=500nm, width w=100nm, placed on (**a**) BK7 glass substrates and (**b**) alumina (Al${}_{2}$O${}_{3}$) substrates of various thickness.

Figure 6 shows the comparison between electric field enhancement and power flow in the cases of BK7 (panel a) or Al${}_{2}$O${}_{3}$ (panel b) used as a substrate of thickness *t*. The results are obtained by varying the thickness of substrate from 0 to 25 nm, with steps of 5 nm. Different electric field enhancement and power flow for different substrate thicknesses are given in Table 3. There is a maximum electric field enhancement at t=5nm, while the power flow reaches its minimum at vanishing substrate thickness.

## 4. Discussion

We have presented a detailed analysis of the optical properties of a gold nanoslit and the effect of two different substrates. The presence of substrate under the nanoslit is used as a tool to control the electric field and power flow. Different parameters influencing the enhancement of the electric field, including size, shape, surrounding medium refractive index and incident wavelength, are discussed.

The RF module in COMSOL Multiphysics was used to perform the numerical simulations of proposed geometry. The width of the gold nanoslit was 100 nm, with a height of 500 nm. The nature of the gold nanoslit was studied using proper boundary conditions. For TM or p-polarization, the findings were stated in terms of the electric field, magnetic field, and power flow. The background electric field was set to 1V/m. In these simulations, a wavelength of 650 nm and a slit width of 100 nm were considered. Based on the results, it is observed that no electric field enhancement occurs for TE or s-polarization. For p-polarization (TM), the reflection and transmission inside the slit generate the Fabry–Perot resonance at the entrance and the exit of the slab when we observe the behavior of the electric field. For the TM mode, regardless of how narrow the slits in the plasmonic slab are, they always support a propagation. In this work, a single wavelength of 650 nm was considered, but these models can be used for broader range of frequencies. The power flow inside the slit decreases when the length of waveguide increases, when using glass and alumina as a substrate the power flow inside the slit decreases.

We considered two different types of substrate, BK7 and Al${}_{2}$O${}_{3}$, investigating their diversified effect on the electric field and power flow in the nanoslit. The case of BK7 provides a more effective electric field enhancement with respect to Al${}_{2}$O${}_{3}$. The comparison in Table 2 and Table 3 shows that one can adjust fields and power flows by using different material combinations for the substrate. In future research, we plan to address more complex geometries relying on the essential nanoslit model presented in this work, with its optical properties, and considering the use of different substrates.

## Figures and Tables

**Table 1:** Parameters of the considered gold nanoslit.

Parameter	Value (nm)
Width of the model	1500
Height of the model	3000
Width of the nanoslit	100
Height of the nanoslit	500
Wavelength	650

**Table 2:** Electric field enhancements and power flows for BK7 substrates of different thicknesses.

BK7 Substrate Thickness	Electric Field Enhancement	Power Flow
(nm)	(V/m)	($10^{-6}\;W/m^{2}$)
0	3.5	2.81–1.50
5	5	2.45–1.30
10	4	1.90–1.05
15	3	1.65–0.90
20	3	1.45–0.75
25	3	1.35–0.65

**Table 3:** Electric field enhancements and power flows for Al${}_{2}$O${}_{3}$ substrates of different thicknesses.

Al${}_{2}$O${}_{3}$ Substrate Thickness	Electric Field Enhancement	Power Flow
(nm)	(V/m)	($10^{-6}\;W/m^{2}$)
0	3.5	2.81–1.50
5	5	1.95–1.15
10	3.5	1.47–0.85
15	3	1.15–0.65
20	2.5	0.97–0.50
25	2.5	0.88–0.40

## Data Availability

Data are partly presented in the manuscript and partly available from the corresponding author on reasonable request.

## Abbreviations

The following abbreviations are used in this manuscript:

FEM	Finite element methods
EOT	Extraordinary optical transmission
SPP	Surface plasmon polaritons
TE	Transverse electric
TM	Transverse magnetic
SP	Surface plasmon
LSPR	Localized surface plasmon resonance
T-SPR	Transmission surface plasmon resonance
